# Comparative characterization of *PCDH19* missense and truncating variants in *PCDH19*-related epilepsy

**DOI:** 10.1038/s10038-020-00880-z

**Published:** 2020-12-02

**Authors:** Mami Shibata, Atsushi Ishii, Ayako Goto, Shinichi Hirose

**Affiliations:** 1 Central Research Institute for the Molecular Pathomechanisms of Epilepsy, Fukuoka University, Fukuoka, Japan; 2 Department of Pediatrics, School of Medicine, Fukuoka University, Fukuoka, Japan

**Keywords:** Genetic association study, Epilepsy, Disease genetics

## Abstract

Missense and truncating variants in protocadherin 19 (*PCDH19*) cause *PCDH19*-related epilepsy. In this study, we aimed to investigate variations in distributional characteristics and the clinical implications of variant type in *PCDH19*-related epilepsy. We comprehensively collected *PCDH19* missense and truncating variants from the literature and by sequencing six exons and intron–exon boundaries of *PCDH19* in our cohort. We investigated the distribution of each type of variant using the cumulative distribution function and tested for associations between variant types and phenotypes. The distribution of missense variants in patients was clearly different from that of healthy individuals and was uniform throughout the extracellular cadherin (EC) domain, which consisted of six highly conserved domains. Truncating variants showed two types of distributions: (1) located from EC domain 1 to EC domain 4, and (2) located from EC domain 5 to the cytoplasmic domain. Furthermore, we also found that later onset seizures and milder intellectual disability occurred in patients with truncating variants located from EC domain 5 to the cytoplasmic domain compared with those of patients with other variants. Our findings provide the first evidence of two types of truncating variants in the *PCDH19* gene with regard to distribution and the resulting clinical phenotype.

## Introduction

Protocadherin 19 (*PCDH19*)-related epilepsy is an epileptic syndrome with various characteristics. Early-onset seizures (6–36 months), which are the most typical characteristic, can be focal, generalized, tonic–clonic, myoclonic, atonic, or absent and often occur in clusters or as prolonged ictal episodes; their severity and frequency vary among affected females, resembling the seizures observed in patients with Dravet syndrome [1–6]. Furthermore, the clinical features of this condition have expanded to include the presence or absence of intellectual disability, psychiatric features, and behavioral disturbances [7–11].

*PCDH19*-related epilepsy is caused by pathogenic variants in the *PCDH19* gene. This gene is located on chromosome Xq22.1 and encodes subgroup of protocadherins, which are involved in signal transduction at synapses and in the establishment of neuronal connections [12]. PCDH19 is a member of the δ2-protocadherin group and contains six extracellular cadherin (EC) repeats, a transmembrane domain, and a cytoplasmic domain, including two conserved motifs. Crystal structural analysis of zebrafish *pcdh19* and solution biophysical resonance analysis of the human δ-protocadherin family have revealed the preferential homophilic trans-interaction of *pcdh19* in the region from EC domain 1 (EC1) to EC domain 4 (EC4) [13, 14]. Moreover, the most supported pathogenic mechanism of *PCDH19*-related epilepsy is mosaicism of two cell populations, expressing either normal or mutant PCDH19 protein [6, 15, 16], which is expected to prevent the normal homophilic adhesive function of PCDH19 proteins.

To date, more than 200 variants have been reported in patients with *PCDH19*-related epilepsy. Most variants (90%) have been identified in female patients, whereas only 10% of variants have been identified in male patients [17]. Of these variants, missense and truncating variants (nonsense and frameshift variants) account for 58% and 41%, respectively, and are observed mostly in exon 1, which encodes all EC domains. Kolc et al. [18] classified these variants according to variant type, location, inheritance, and clinical features, including seizure onset age and intellectual disability, and examined genotype–phenotype associations. They found that earlier seizure onset (≤12 months) was significantly associated with more severe intellectual disability, whereas cognitive function did not differ between variant types for missense and truncating variants, between variant locations, from EC1 to EC3, and EC4 to the cytoplasmic domain. Currently, no genotype–phenotype correlations have been identified.

In this study, we updated pathogenic variants by collecting data from published and unpublished reports of patients with *PCDH19*-related epilepsy. We also evaluated differences in variant location and clinical phenotypes among variant types. By analyzing the variant distribution using the cumulative distribution function (CDF), we found that truncating variants in the *PCDH19* gene could be separated into two types: (1) located from EC1 to EC4 and (2) located from EC domain 5 to the cytoplasmic domain. Furthermore, a comparison of clinical phenotypes revealed the occurrence of early-onset seizure and milder intellectual disability in patients with the second type of truncating variant compared with that in patients carrying missense variants and the first type of truncating variant.

## Materials and methods

### Patients and clinical information

Our patients, who were diagnosed with *PCDH19*-related epilepsy, satisfied the International League Against Epilepsy definition (https://www.epilepsydiagnosis.org/aetiology/gene-abnormalities-overview.html#PCDH19), as follows: (1) seizures starting early (mean age 9 months); (2) tonic–clonic and/or focal seizures; (3) frequent, clustered seizures facilitated by fever; and (4) intellectual development that varied from normal, often with regression, to severe intellectual disability with autistic features and psychiatric disorders.

We collected clinical information using a questionnaire with an organized form, including perinatal history, family history, age at seizure onset, seizure history, psychomotor development, behavioral features, intellectual disability level, neuroimaging findings, electroencephalographic findings, and treatment. After checking clinical information, patients whose parents provided signed informed consent using a protocol approved by the ethics review committee of Fukuoka University underwent genetic analyses.

### Identification of *PCDH19* pathogenic variants

The presence of *PCDH19* gene variants in the above patients was evaluated by Sanger or gene panel sequencing [19–21]. Gene panel sequencing was performed using a customized HaloPlex Target Enrichment System (Agilent, Santa Clara, CA, USA) for 114 genes that are known or suspected to cause epileptic seizures. Samples were sequenced on a MiSeq instrument (Illumina, San Diego, CA, USA). The exon regions of targeted genes were covered with ~100 reads on average. Variants were called from sequencing results using SureCall software (v4.0, Agilent, Santa Clara, CA, USA). Using ANNOVAR software [22], the pathogenicity of variants was estimated based on allele frequencies of 621 East Asian individuals in 1000 Genomes Project data (published in 2015, https://www.internationalgenome.org/), 9977 East Asian individuals in gnomAD (v2.1.1, https://www.internationalgenome.org/; the Genome Aggregation Database [gnomAD], https://gnomad.broadinstitute.org/), and 4456 Japanese individuals in the Human Genetic Variation database (v2.3, Human Genetic Variation Database in the Japanese population, http://www.hgvd.genome.med.kyoto-u.ac.jp/ [23]) and by nucleotide or amino acid conservation and their effects on protein structure predicted by SIFT, PolyPhen2, Mutation Taster, CADD, and PhyloP100way vertebrate. Variants with an allele frequency ≥0.005 in these databases were considered nonpathogenic variants. Furthermore, variants with CADD ≥15 and/or with PhyloP100way vertebrate score ≥3, as well as variants predicted to be deleterious by SIFT, PolyPhen2, and Mutation Taster were preferentially considered pathogenic variants. To investigate large deletions or duplications, multiplex ligation-dependent probe amplification (MLPA) was performed using SALSA MLPA KIT P330-A3 PCDH19 (MRC-Holland, Amsterdam, the Netherlands). When a pathogenic variant was suspected, inheritance was determined by sequencing or by MLPA for available parental DNA.

### Collection of *PCDH19* variants and clinical information from the literature

For this study, *PCDH19* variants were comprehensively gathered from the EpilepsyGene database (accessed on January 6, 2017; http://61.152.91.49/EpilepsyGene/index.php) and from Google Scholar, searched using the words “PCDH19” and “epilepsy” (accessed on May 29, 2020; https://scholar.google.co.jp/). To avoid the risk of bias by overcollection, we adopted the variant and clinical information for one case per family. For collection of intellectual disability information, we adopted only intellectual disability information following the classification of normal, borderline, mild, moderate, severe, or profound, according to the previously established criteria [18].

### Statistical analysis

For CDF analysis, we used the coding sequence (CDS) position (transcript ID: NM_001184880.2) of missense and truncating variants (frameshift and nonsense variants) reported in female patients. In CDF analysis, variant distributions were assessed by Anderson–Darling tests, with the hypothesis that the distribution was uniform along the CDS position of *PCDH19*. The difference in seizure onset age between variant types was assessed by Kruskal–Wallis test and pair-wise Wilcoxon tests with Bonferroni multiple comparison correction, excluding abnormal onset age (more than 36 months). The proportions of patients with each type of clinical feature were compared using pair-wise chi-square tests with Bonferroni multiple comparison correction. The difference in the rate of patient with each intellectual disability level was assessed by hierarchical clustering analysis with Ward’s method and Euclidean distance. These statistical calculations were performed using R software (version 3.6.0), and results with *p* values of less than 0.05 were considered significant.

## Results

### Collection of *PCDH19* variants

We collected 455 *PCDH19* pathogenic variants and clinical features from 13 Japanese patients in our cohort (Supplementary Table 1) and from 442 patients in the literature (Supplemental Table 2). Of these variants, 427 variants (93.8%) were reported in females, and 28 variants (6.15%) were reported in males. The variant types and inheritances were summarized according to sex (Table 1). For both sexes, most commonly reported variants were missense, frameshift, and nonsense variants (198 [46.4%: 198/427], 109 [25.5%: 109/427], and 64 [15.0%: 64/427] in females; 13 [46.4%: 13/28], 3 [10.7%: 3/28], and 9 [32.1%: 9/28] in males). Furthermore, most of these missense, frameshift, and nonsense variants were de novo in both sexes (99 [50.0%: 99/198], 66 [60.6%: 66/109], and 33 [51.6%: 33/64] in females; 8 [61.5%: 8/13], 2 [66.7%: 2/3], 7 [77.8%: 7/9] in males).

**Table 1:** Summary of variant types and inheritance by sex

	Female						Male					
	De novo	Paternal	Maternal	Familial	Unknown	Total	De novo	Paternal	Maternal	Familial	Unknown	Total
Missense	99	35	32	1	31	198	8	0	0	0	5	13
Compound heterozygous missense	4	1	0	0	0	5	0	0	0	0	0	0
Frameshift	66	16	4	4	19	109	2	0	1	0	0	3
Nonsense	33	9	4	6	12	64	7	0	0	0	2	9
Splicing	8	0	1	0	5	14	2	0	0	0	0	2
Inframe-insertion	1	0	2	0	0	3	0	0	0	0	0	0
Inframe-deletion	2	0	0	0	0	2	0	0	0	0	0	0
Inframe-insertion/deletion	1	0	0	0	2	3	0	0	0	0	0	0
Microdeletion	2	0	1	0	1	4	0	0	0	0	0	0
Microduplication	1	0	0	0	0	1	0	0	0	0	0	0
Whole gene deletion	16	1	0	0	7	24	1	0	0	0	0	1
Total	233	62	44	11	77	427	20	0	1	0	7	28

Of 28 male patients and 62 transmitting fathers of female patients, mosaic and hemizygous males comprised 27 and 12 cases, respectively (Supplementary Tables 1 and 2). Most mosaic males (88.9%: 24/27) showed seizure-related features, whereas, 83.3% (10/12) of hemizygous males were unaffected, in accordance with previously suggested pathomechanisms of *PCDH19*-related epilepsy [6, 15, 16].

### Distribution analysis of *PCDH19* variants

Of the reported variants, we used the most commonly reported variants, i.e., missense, frameshift, and nonsense variants, in female patients. In particular, frameshift and nonsense variants were grouped into truncating variants, which generate a premature stop codon, and 198 missense and 173 truncating variants (109 frameshift and 64 nonsense variants) were used for distribution analysis.

**Fig. 1:**
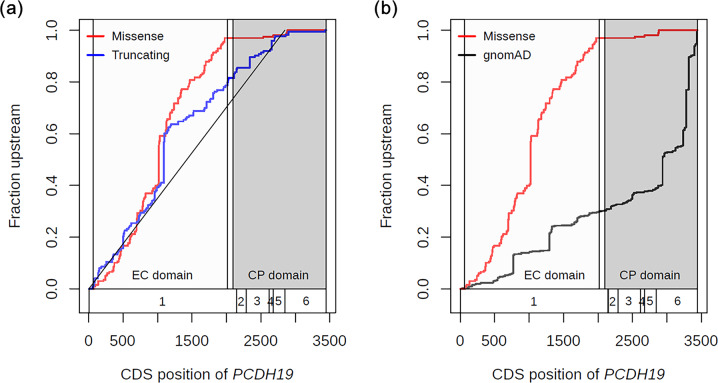
CDFs of *PCDH19* missense and truncating variants. **a** CDFs of missense (red line) and truncating variants (blue line). **b** CDFs of variants from the gnomAD database (black line) and missense variants (red line). The white boxes and numbers at the bottom of the graphs indicate exon regions and exon numbers for the *PCDH19* gene. The light and dark gray regions indicate extracellular cadherin (EC) and cytoplasmic (CP) domains, respectively

First, we compared the distributions of missense and truncating variants using the CDF (Fig. 1). The CDFs showed extremely high frequencies (more than 20 cases) at CDS positions 1019 and 1091 for missense variant (NM_001184880.2:c.1019A>G [chrX.hg19:g.99662577T>C]) and truncating variants (NM_001184880.2:c.1091dupC [chrX.hg19:g.99662505dupG] and NM_001184880.2:c.1091delC [chrX.hg19:g.99662505delG]; Fig. 1a), respectively. The numbers of patients carrying these missense variant (NM_001184880.2:c.1019A>G) and truncating variants (NM_001184880.2:c.1091dupC and c.1091delC) were 30, 22, and 5, respectively (Supplementary Tables 1 and 2). Furthermore, very low frequencies of missense and truncating variants were observed in the cytoplasmic domain and in the last exon, respectively.

In order to investigate whether variants located in the cytoplasmic domain region were lethal or noncausal variants of *PCDH19*-related epilepsy, we compared the CDFs of missense variants with those of variants from healthy individuals in gnomAD database, including various ethnic backgrounds (Fig. 1b). As a result, the distribution of missense variants did not overlap with that of healthy individual variants in any ethnic background, except with seven positions. The maximum allele frequencies of healthy variants at the seven positions were less than 0.005 (0–0.00208). Furthermore, the CDFs of gnomAD database variants showed high frequencies in the cytoplasmic domain and low frequencies in the EC domains, in contrast with the CDFs of missense variants. These results suggested that most missense variants in the cytoplasmic domain were not lethal and did not cause *PCDH19*-related epilepsy.

**Fig. 2:**
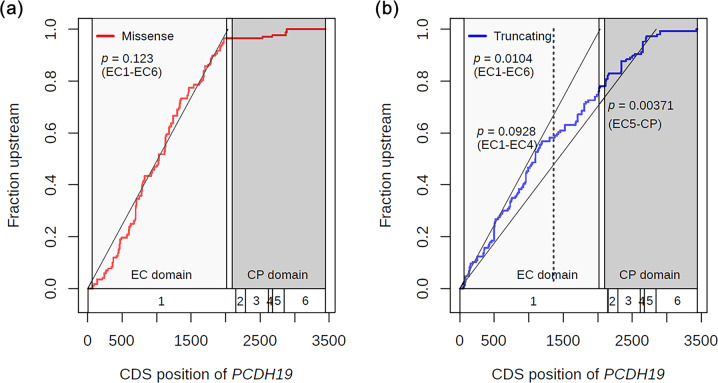
CDFs of *PCDH19* missense and truncating variants without hotspots. **a** CDFs of missense variants without hotspots (red line). **b** CDFs of truncating variants without hotspots (blue line). The white boxes and numbers at the bottom of the graphs indicate exon regions and exon numbers for the *PCDH19* gene. The light and dark gray regions indicate extracellular cadherin (EC) and cytoplasmic (CP) domains, respectively. The dashed line indicates the boundary between EC domains 4 and 5

After exclusion of high-frequency variants, we further investigated the uniformity of the distributions of missense and truncating variants using Anderson–Darling tests. The results showed that the distribution of missense variants was uniform throughout the EC domain (*p* = 0.123, Fig. 2a). In contrast, the distribution of truncating variants was not uniform throughout the EC domain (*p* = 0.0104, Fig. 2b). As a result of more detailed Anderson–Darling tests, the distribution of truncating variants was found to be uniform from EC1 to EC4 (*p* = 0.0928, Fig. 2b, Supplementary Table 3). These results indicated that there were at least two types of truncating variants in the *PCDH19* gene, i.e., variants located from EC1 to EC4 and variants located from EC5 to the cytoplasmic domain. In addition, truncating variants were not uniform from the start codon to the region upstream of the last exon (*p* = 0.00371, Fig. 2b).

### Patient phenotypes associated with each variant type

CDF analysis revealed that *PCDH19* variants could be classified into three types: (1) missense variants, (2) truncating variants located from EC1 to EC4, and (3) truncating variants located from EC5 to the cytoplasmic domain. Next, we assessed the phenotypic differences of patients with *PCDH19*-related epilepsy, including the age of seizure onset, seizure type, behavioral features, and intellectual disability level, according to variant type using clinical data. In addition, we classified truncating variants as (1) those located from EC1 to EC3 and (2) those located from EC4 to cytoplasmic domain [18] and then assessed phenotypic differences among patients with variant types.

**Fig. 3:**
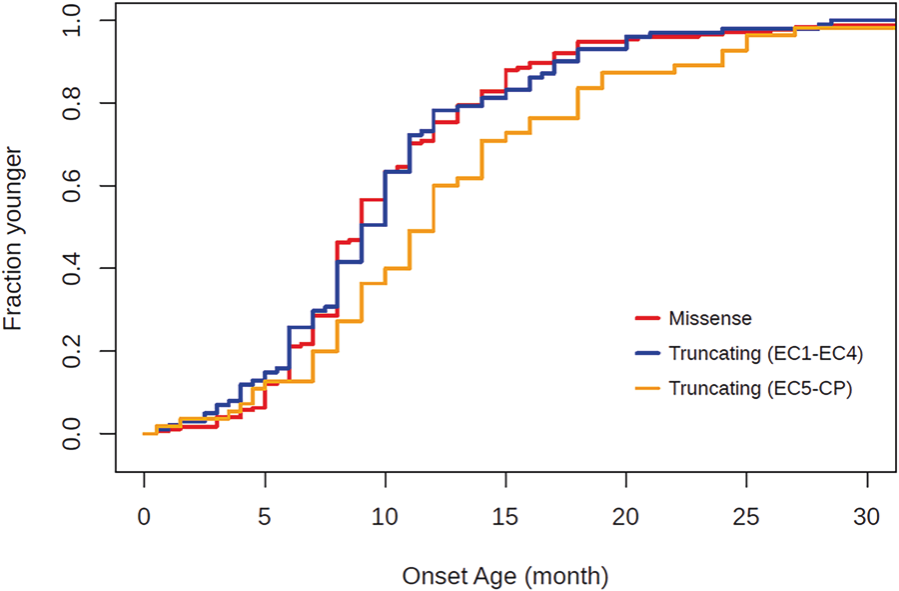
CDFs of seizure onset age. CDFs of the onset age of patients with missense variants, truncating variants located from EC1 to EC4, and truncating variants located from EC5 to the cytoplasmic domain are indicated in red, blue, and orange, respectively

First, we investigated the age at onset of seizures in patients with each variant type (Fig. 3). Of the collected clinical features, seizure onset age under 36 months was observed for 175, 101, and 55 patients carrying missense variants, truncating variants located from EC1 to EC4, and truncating variants located from EC5 to the cytoplasmic domain, respectively. The mean age of patients with missense variants was 10.2 months (standard deviation [SD]: 5.26 months), and those in patients with truncating variants from EC1 to EC4 and from EC5 to the cytoplasmic domain were 10.1 months (SD: 5.37 months) and 12.6 months (SD: 6.66 months), respectively, indicating that the mean ages of patients with truncating variants located from EC5 to the cytoplasmic domain were ~2.44 and 2.54 months older than those of patients with the other types of variants. Furthermore, there were significant differences between truncating variants located from EC5 to the cytoplasmic domain and the other variant types (*p* = 0.0152 [Kruskal–Wallis test], and *p* = 0.0173 and 0.0354 [missense versus truncating variants located from EC5 to the cytoplasmic domain and truncating variants located from EC1 to EC4 versus truncating variants located from EC5 to the cytoplasmic domain; pair-wise Wilcoxon tests]). In addition, the mean ages of patients with truncating variants located from EC1 to EC3 and of patients with truncating variants from EC4 to the cytoplasmic domain were 10.9 months (SD: 5.84 months) and 11.0 months (SD: 6.07 months), respectively. There were no significant difference among missense variants and these truncating variant types (*p* = 0.375 [Kruskal–Wallis test], and *p* = 0.887, 0.691, and 1.00 [missense versus truncating variants located from EC1 to EC3, missense versus truncating variants from EC4 to cytoplasmic domain, and truncating variants located from EC1 to EC3 versus truncating variants located from EC4 to the cytoplasmic domain, respectively; pair-wise Wilcoxon tests], Supplementary Fig. 1).

Next, we assessed nine seizure-related features, including “febrile”, “tonic–clonic”, “myoclonic”, “complex partial seizure/absence”, and “status”, as well as behavioral features, including autistic and behavioral disturbances, in patients with *PCDH19*-related epilepsy (Table 2). Seizure type data were available in 149, 85, and 43 patients with missense variants, truncating variants located from EC1 to EC4, and truncating variants located from EC5 to the cytoplasmic domain, respectively. Behavioral feature data were available for 150, 85, and 43 patients with missense variants, truncating variants located from EC1 to EC4, and truncating variants located from EC5 to the cytoplasmic domain. Of the seizure-related features, high-rate features included “cluster” (65.9–83.7%), “febrile seizures” (55.3–69.8%), “focal seizures” (64.7–67.8%), and “tonic–clonic seizures” (45.9–53.5%), for all variant types. All clinical features showed no significant differences between variant types (*p* = 0.149–1.00). Similar to seizure-related features, the percentages of patients with autistic features and/or behavioral disturbance were also high (51.2–64.7%) for all variant types and did not differ significantly between variant types (*p* = 0.594–1.00, Table 2). Furthermore, there were also no significant differences in these clinical features among missense variants, truncating variants located from EC1 to EC3, and truncating variants located from EC4 to the cytoplasmic domain (*p* = 0.142–1.00, Supplementary Table 4).

**Table 2:** Summary of seizure types and behavioral features

Seizure-related features	Missense (total available data: 149)		Truncating (EC1–EC4) (total available data: 85)		Truncating (EC5–CP) (total available data: 43)		*p* values (missense vs truncating (EC1–EC4))	*p* values (missense vs truncating (EC5–CP))	*p* values (truncating (EC1–EC4) vs truncating (EC5–CP))
	Patient number	%	Patient number	%	Patient number	%			
Febrile seizures	87	58.4	47	55.3	30	69.8	1.00	0.726	0.495
Tonic-clonic seizures	72	48.3	39	45.9	23	53.5	1.00	1.00	1.00
Tonic seizures	43	28.9	22	25.9	13	30.2	1.00	1.00	1.00
Hemiclonic/Unilat	12	8.05	5	5.88	2	4.65	1.00	1.00	1.00
Myoclonic seizures	12	8.05	8	9.41	2	4.65	1.00	1.00	1.00
Complex partial seizure/absence	26	17.4	14	16.5	6	14.0	1.00	1.00	1.00
Partial(Focal) seizures	101	67.8	55	64.7	28	65.1	1.00	1.00	1.00
Status epilepticus	29	19.5	10	11.8	9	20.9	0.543	1.00	0.795
Cluster	117	78.5	56	65.9	36	83.7	0.149	1.00	0.168

Behavioral features	Missense (total available data: 150)		Truncating (EC1–EC4) (total available data: 85)		Truncating (EC5–CP) (total available data: 43)		*p* values (missense vs truncating (EC1–EC4))	*p* values (missense vs truncating (EC5–CP))	*p* values (truncating (EC1–EC4) vs truncating (EC5–CP))
	Patient number	%	Patient number	%	Patient number	%			
Autistic features/behavioral disturbances	87	58.0	55	64.7	22	51.2	1.00	1.00	0.594

*p* values were calculated by pair-wise chi-squared tests and corrected by Bonferroni multiple correction. “EC” and “CP” indicate “extracellular cadherin” and “cytoplasmic”, respectively

**Fig. 4:**
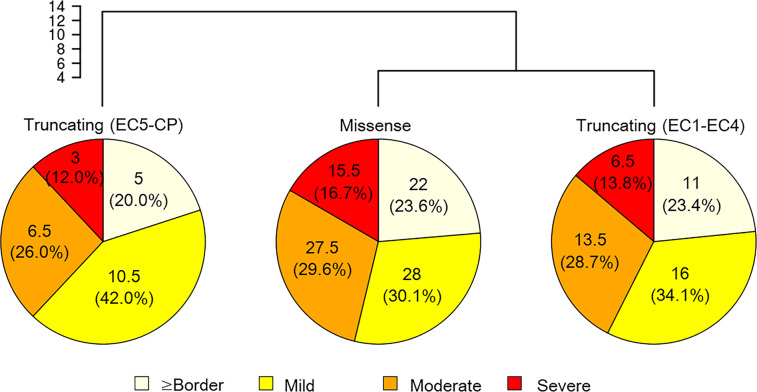
Comparison of the level of intellectual disability of in patients with *PCDH19*-related epilepsy. Hierarchical clustering dendrogram (top) showing similarities in the rates of patients with each intellectual disability level. For clustering, Ward’s method and Euclidean distance were utilized. The pie charts (bottom) show the percentages of patients with each level of intellectual disability. Cases with two levels in the literature were counted as 0.5 cases. For example, one case with a “moderate/severe” level was counted as 0.5 cases of moderate level and 0.5 cases of severe level

Finally, differences in intellectual disability levels between variant types were assessed using pie charts and clustering analysis based on the numbers and rates of patients with each intellectual disability level (Fig. 4). The intellectual disability levels of patients were available for 93 missense variants, 47 truncating variants located from EC1 to EC4, and 25 truncating variants located from EC5 to the cytoplasmic domain. The most common intellectual disability level was “mild” for all variant types (30.1–42.0%). Furthermore, the total percentage of patients with “≥ border” and “mild” intellectual disability levels was highest in patients with truncating variants located from EC5 to the cytoplasmic domain (62.0%), compared with those in patients with missense variants and truncating variants located from EC1 to EC4 (53.7% and 57.5%, respectively). Although there were no statistically significant differences (*p* = 1.00), these results suggested that the severity of intellectual disability in patients tends to be milder in patients with truncating variants located from EC5 to the cytoplasmic domain than in patients with other variant types. Furthermore, clustering analysis showed results consistent with those from pie charts. Hierarchical clustering dendrograms demonstrated a higher degree of similarity for smaller distances. The distances between truncating variants located from EC5 to the cytoplasmic domain and the other variant types were 13.8 and 9.26, whereas the distance between missense and truncating variants located from EC1 to EC4 was 4.93, clustering in one group. Taken together, these results suggested that the intellectual disability level of patients with truncating variants located from EC5 to the cytoplasmic domain was separated from those of patients with other variant types. In contrast, although the intellectual disability levels of missense and truncating variants from EC1 to EC3 were classified into one group in the clustering analysis, the distances were high and did not show major differences (7.75–10.5) among missense variants, truncating variants located from EC1 to EC3, and truncating variants located from EC4 to the cytoplasmic domain. These results indicated the differences of intellectual disability levels among patients with these variant types (Supplementary Fig. 2).

## Discussion

In this study, we investigated the distributional characteristics and clinical implications of each type of *PCDH19* pathogenic variant.

Distribution analysis revealed very high frequencies (more than 20 cases) at two CDS positions, 1019 and 1091, for missense and frameshift variants (NM_001184880.2:c.1019A>G, c.1091dupC, and c.1091delC). Of these variants, missense variant (NM_001184880.2:c.1019A>G) and frameshift variant (NM_001184880.2:c.1091dupC) have been called “hotspots” or “recurrent” variants [17, 18]. Focusing on the CDS of *PCDH19* mRNA (NM_001184880.2), we found mononucleotide repeats (seven cytosine nucleotide repeats) in the upstream region of the frameshift hotspot variant. Mononucleotide repeats have been founded in gastric and colorectal cancers and are considered targets of frameshift variants [24–29**]**. Therefore, the seven cytosine nucleotide repeats may be a cause of frameshift hotspot variants. However, there were no mononucleotide repeats or other causal sequences, such as CG-rich sequences, at nearby missense hotspot variants. The occurrence of missense hotspot variants may be due to some external factors, such as the high susceptibility of codons to variants and slow DNA repair rates [30].

After exclusion of hotspot variants, our analysis revealed that although missense variants were distributed uniformly throughout EC domain, truncating variants could be separated into at least two types: (1) located from EC1 to EC4 and (2) located from EC5 to the cytoplasmic domain. Previous our studies have observed that truncating variants distributed uniformly from the start codon to 50 nt upstream region of the last exon in some epileptic genes [20, 21**]**. Therefore, this non-uniformity of the distribution indicates the specificity of truncating variants in the *PCDH19* gene.

In support of this result, we found differences in seizure onset age and intellectual disability levels between the second type of truncating variants and the other variant types. The mean age of seizure onset of patients carrying the second type of truncating variants (about 12 months) was 2 months older than those of patients carrying the other variant types (about 10 months). Furthermore, the intellectual disability of patients carrying the second type of truncating variants was milder than those of patients carrying the other variant types. Notably, these results were concordant with the observations of Kolc et al. [18], who showed that earlier seizure onset (≤12 months) was significantly associated with more severe intellectual disability. Furthermore, they also found that there was no association between patient phenotype and variant location following the classification of variants located from EC1 to EC3, and variants located from EC4 to cytoplasmic domain. Our truncating variant data showed the association between patient phenotypes (seizure onset age and intellectual disability) and variant location (located from EC1 to EC4 and located from EC5 to the cytoplasmic domain), whereas no association was observed when using their classification. Therefore, whether the variant is located upstream of EC5 or not may be crucial for determination of phenotypic severity with regard to seizure onset age and intellectual disability in patients with *PCDH19*-related epilepsy carrying truncating variants.

Recent crystal structure analysis of zebrafish *pcdh19* and surface plasmon resonance data revealed that domains EC1 to EC4 of Pcdh19 encompass the minimal unit of its homophilic adhesive function and that missense mutations in conserved regions of these EC domains abolish the homophilic binding of Pcdh19 [13, 14]. However, the effect of truncating variants on the adhesive function of PCDH19 protein remains unclear. Truncating variants result often contain premature termination codons (PTCs), most of which trigger a process called nonsense-mediated mRNA decay (NMD), thereby preventing the synthesis of truncated and potentially toxic proteins [31–33]. A previous study reported that a PTC-bearing *PCDH19* mRNA was downregulated in skin fibroblast cells by NMD [15]. Furthermore, there are no reports on the existence of truncated PCDH19 proteins in human cells. Thus, further studies are needed to elucidate the pathomechanisms underlying the differential phenotypes in patients harboring different types of truncating variants.

In conclusion, we found that variant types in the *PCDH19* gene could be separated into three types: missense variants, truncating variants located from EC1 to EC4, and truncating variants located from EC5 to the cytoplasmic domain. In addition, variant distribution, seizure onset age, and severity of intellectual disability in patients were similar among patients with missense and truncating variants located from EC1 to EC4, whereas patients carrying truncating variants located from EC5 to the cytoplasmic domain had later onset seizures and milder intellectual disability. Other epilepsy-causative genes, *SCN1A* and *SCN2A*, which encode the voltage-gated sodium channels type 1 and 2, respectively, have shown phenotypic differences based on variant types. As for the *SCN1A* gene, it has been proposed that compared with those with missense variants, truncating variants result in earlier onset of seizures and more severe phenotypes [34, 35]. Our *PCDH19* gene analysis showed similar phenotypes between missense and truncating variants from EC1 to EC4 and showed milder phenotypes with *PCDH19*-truncating variants from EC5 to the cytoplasmic domain. As for the *SCN2A* gene, it has shown differential phenotypes within missense variants, which could be classified into two groups, namely gain-of-function and loss-of-function variants. The gain-of-function variants are mostly located at the voltage sensor domain of the channel and cause infantile epileptic encephalopathy and benign infantile seizures. Meanwhile, the loss-of-function variants are mostly located at the pore loop regions and are associated with autism spectrum disorder and/or intellectual disability, resulting in a variety of seizure types [36–38**]**. *PCDH19* variant analysis showed locational and phenotypic differences within truncating variants. To date, no other reports have described similar genotype–phenotype associations with the *PCDH19* gene. Our findings could contribute to diagnosis of *PCDH19*-related epilepsy and could help to elucidate the pathogenic mechanisms of this disease. For more accurate evaluation of patient phenotypes and understanding of pathogenic mechanisms, we will investigate the detailed effects of transcript or protein products generated from each variant type in our future work.

## Supplementary information

Supplemental Figure 1

Supplemental Figure 2

Supplemental Table 1

Supplemental Table 2

Supplemental Table 3

Supplemental Table 4

Supplemental References
