# Supplementary figures and images for: Comparative characterization of PCDH19 missense and truncating variants in PCDH19-related epilepsy
Source: J Hum Genet. 2020 Dec 2;66(6):569–78. doi: 10.1038/s10038-020-00880-z (PMC8144015; doi:10.1038/s10038-020-00880-z)

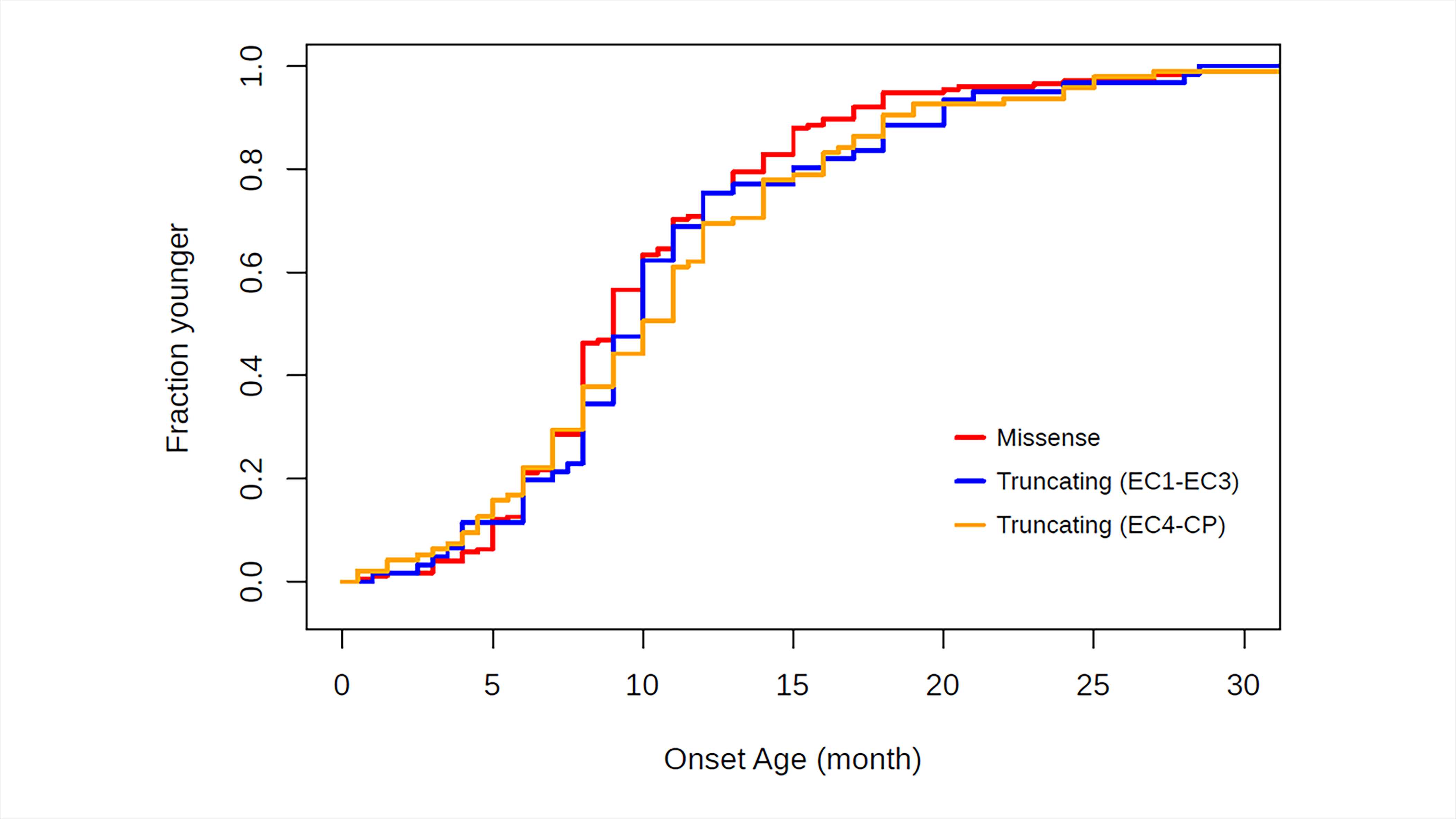

Supplement: Supplementary file 1 — Supplemental Figure 1 [file 10038_2020_880_MOESM1_ESM.tif]

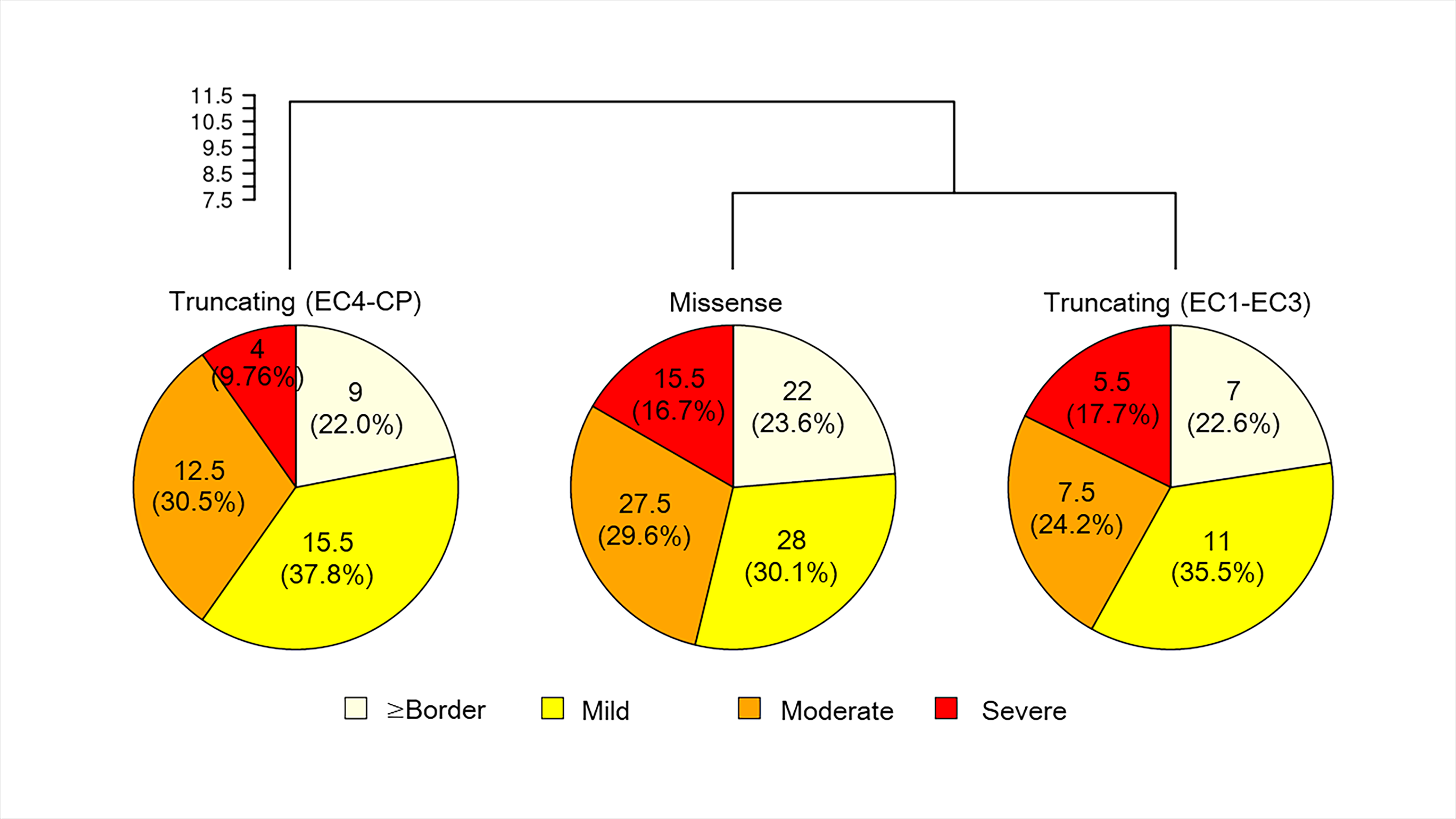

Supplement: Supplementary file 2 — Supplemental Figure 2 [file 10038_2020_880_MOESM2_ESM.tif]
