# Supplementary material for: Comparative characterization of PCDH19 missense and truncating variants in PCDH19-related epilepsy
Source: J Hum Genet. 2020 Dec 2;66(6):569–78. doi: 10.1038/s10038-020-00880-z (PMC8144015; doi:10.1038/s10038-020-00880-z)
Supplement: Supplementary file 7 — Supplemental References [file 10038_2020_880_MOESM7_ESM.docx]

**Supplemental References**

The literatures reported *PCDH19* variants in Supplemental Table 2.

1. Breuillard D, Leunen D, Chemaly N, Auclair L, Pinard JM, Kaminska A, et al. Autism spectrum disorder phenotype and intellectual disability in females with epilepsy and PCDH-19 mutations. Epilepsy Behav. 2016;60:75-80.

2. Camacho A, Simon R, Sanz R, Vinuela A, Martinez-Salio A, Mateos F. Cognitive and behavioral profile in females with epilepsy with PDCH19 mutation: two novel mutations and review of the literature. Epilepsy Behav. 2012;24(1):134-7.

3. Cappelletti S, Specchio N, Moavero R, Terracciano A, Trivisano M, Pontrelli G, et al. Cognitive development in females with PCDH19 gene-related epilepsy. Epilepsy Behav. 2015;42:36-40.

4. Carvill GL, Heavin SB, Yendle SC, McMahon JM, O'Roak BJ, Cook J, et al. Targeted resequencing in epileptic encephalopathies identifies de novo mutations in CHD2 and SYNGAP1. Nat Genet. 2013;45(7):825-30.

5. de Lange IM, Rump P, Neuteboom RF, Augustijn PB, Hodges K, Kistemaker AI, et al. Male patients affected by mosaic PCDH19 mutations: five new cases. neurogenetics. 2017;18(3):147-53.

6. Depienne C, Bouteiller D, Keren B, Cheuret E, Poirier K, Trouillard O, et al. Sporadic infantile epileptic encephalopathy caused by mutations in PCDH19 resembles Dravet syndrome but mainly affects females. PLoS Genet. 2009;5(2):e1000381.

7. Depienne C, Trouillard O, Bouteiller D, Gourfinkel-An I, Poirier K, Rivier F, et al. Mutations and deletions in PCDH19 account for various familial or isolated epilepsies in females. Hum Mutat. 2011;32(1):E1959-75.

8. Depienne C, LeGuern E. PCDH19-related infantile epileptic encephalopathy: an unusual X-linked inheritance disorder. Hum Mutat. 2012;33(4):627-34.

9. Dibbens LM, Tarpey PS, Hynes K, Bayly MA, Scheffer IE, Smith R, et al. X-linked protocadherin 19 mutations cause female-limited epilepsy and cognitive impairment. Nat Genet. 2008;40(6):776-81.

10. Dibbens LM, Kneen R, Bayly MA, Heron SE, Arsov T, Damiano JA, et al. Recurrence risk of epilepsy and mental retardation in females due to parental mosaicism of PCDH19 mutations. Neurology. 2011;76(17):1514-9.

11. Hynes K, Tarpey P, Dibbens LM, Bayly MA, Berkovic SF, Smith R, et al. Epilepsy and mental retardation limited to females with PCDH19 mutations can present de novo or in single generation families. J Med Genet. 2010;47(3):211-6.

12. Ryan SG, Chance PF, Zou CH, Spinner NB, Golden JA, Smietana S. Epilepsy and mental retardation limited to females: an X-linked dominant disorder with male sparing. Nat Genet. 1997;17(1):92-5.

13. Juberg RC, Hellman CD. A new familial form of convulsive disorder and mental retardation limited to females. The Journal of pediatrics. 1971;79(5):726-32.

14. Fabisiak K, Erickson RP. A familial form of convulsive disorder with or without mental retardation limited to females: extension of a pedigree limits possible genetic mechanisms. Clin Genet. 1990;38(5):353-8.

15. Dimova PS, Kirov A, Todorova A, Todorov T, Mitev V. A novel PCDH19 mutation inherited from an unaffected mother. Pediatr Neurol. 2012;46(6):397-400.

16. Gagliardi M, Annesi G, Sesta M, Tarantino P, Conti P, Labate A, et al. PCDH19 mutations in female patients from Southern Italy. Seizure. 2015;24:118-20.

17. Gursoy S, Ataman E, Baysal BT, Özyılmaz B, Genҫpınar P, Hız AS, et al. Identification of *PCDH19* gene mutations/deletions in patients with early onset epilepsy. Annals of Indian Academy of Neurology. 2020;23(2):206-10.

18. Higurashi N, Shi X, Yasumoto S, Oguni H, Sakauchi M, Itomi K, et al. PCDH19 mutation in Japanese females with epilepsy. Epilepsy Res. 2012;99(1-2):28-37.

19. Higurashi N, Nakamura M, Sugai M, Ohfu M, Sakauchi M, Sugawara Y, et al. PCDH19-related female-limited epilepsy: further details regarding early clinical features and therapeutic efficacy. Epilepsy Res. 2013;106(1-2):191-9.

20. Higurashi N, Takahashi Y, Kashimada A, Sugawara Y, Sakuma H, Tomonoh Y, et al. Immediate suppression of seizure clusters by corticosteroids in PCDH19 female epilepsy. Seizure. 2015;27:1-5.

21. Homan CC, Pederson S, To T-H, Tan C, Piltz S, Corbett MA, et al. PCDH19 regulation of neural progenitor cell differentiation suggests asynchrony of neurogenesis as a mechanism contributing to PCDH19 Girls Clustering Epilepsy. Neurobiology of Disease. 2018;116:106-19.

22. Jamal SM, Basran RK, Newton S, Wang Z, Milunsky JM. Novel de novo PCDH19 mutations in three unrelated females with epilepsy female restricted mental retardation syndrome. Am J Med Genet A. 2010;152A(10):2475-81.

23. Kolc KL, Sadleir LG, Depienne C, Marini C, Scheffer IE, Møller RS, et al. A standardized patient-centered characterization of the phenotypic spectrum of PCDH19 girls clustering epilepsy. Translational Psychiatry. 2020;10(1):127.

24. Kurian M, Korff CM, Ranza E, Bernasconi A, Lübbig A, Nangia S, et al. Focal cortical malformations in children with early infantile epilepsy and PCDH19 mutations: case report. Developmental Medicine & Child Neurology. 2018;60(1):100-5.

25. Kwong AK, Fung CW, Chan SY, Wong VC. Identification of SCN1A and PCDH19 mutations in Chinese children with Dravet syndrome. PLoS One. 2012;7(7):e41802.

26. Kwong AK, Ho AC, Fung CW, Wong VC. Analysis of mutations in 7 genes associated with neuronal excitability and synaptic transmission in a cohort of children with non-syndromic infantile epileptic encephalopathy. PLoS One. 2015;10(5):e0126446.

27. Leonardi E, Sartori S, Vecchi M, Bettella E, Polli R, Palma LD, et al. Identification of four novel PCDH19 Mutations and prediction of their functional impact. Ann Hum Genet. 2014;78(6):389-98.

28. Liu A, Xu X, Yang X, Jiang Y, Yang Z, Liu X, et al. The clinical spectrum of female epilepsy patients with PCDH19 mutations in a Chinese population. Clin Genet. 2017;91(1):54-62.

29. Liu A, Yang X, Yang X, Wu Q, Zhang J, Sun D, et al. Mosaicism and incomplete penetrance of *PCDH19* mutations. Journal of Medical Genetics. 2019;56(2):81.

30. Lyons S, Marnane M, Reavey E, Williams N, Costello D. PCDH19-related epilepsy: a rare but recognisable clinical syndrome in females. Practical Neurology. 2017;17(4):314.

31. Marini C, Mei D, Parmeggiani L, Norci V, Calado E, Ferrari A, et al. Protocadherin 19 mutations in girls with infantile-onset epilepsy. Neurology. 2010;75(7):646-53.

32. Park YN, Jang M-A, Park S. A Novel Pathogenic Variant (c.592_599del) in PCDH19 in a Korean Family with Epilepsy. Ann Child Neurol. 2019;27(4):152-4.

33. Perez D, Hsieh DT, Rohena L. Somatic Mosaicism of PCDH19 in a male with early infantile epileptic encephalopathy and review of the literature. American Journal of Medical Genetics Part A. 2017;173(6):1625-30.

34. Perucca P, Scheffer IE, Harvey AS, James PA, Lunke S, Thorne N, et al. Real-world utility of whole exome sequencing with targeted gene analysis for focal epilepsy. Epilepsy Research. 2017;131:1-8.

35. Romasko EJ, DeChene ET, Balciuniene J, Akgumus GT, Helbig I, Tarpinian JM, et al. PCDH19-related epilepsy in a male with Klinefelter syndrome: Additional evidence supporting PCDH19 cellular interference disease mechanism. Epilepsy Research. 2018;145:89-92.

36. Sadleir LG, Kolc KL, King C, Mefford HC, Dale RC, Gecz J, et al. Levetiracetam efficacy in PCDH19 Girls Clustering Epilepsy. European Journal of Paediatric Neurology. 2020;24:142-7.

37. Scheffer IE, Turner SJ, Dibbens LM, Bayly MA, Friend K, Hodgson B, et al. Epilepsy and mental retardation limited to females: an under-recognized disorder. Brain. 2008;131(Pt 4):918-27.

38. Smith L, Singhal N, El Achkar CM, Truglio G, Rosen Sheidley B, Sullivan J, et al. PCDH19-related epilepsy is associated with a broad neurodevelopmental spectrum. Epilepsia. 2018;59(3):679-89.

39. Specchio N, Marini C, Terracciano A, Mei D, Trivisano M, Sicca F, et al. Spectrum of phenotypes in female patients with epilepsy due to protocadherin 19 mutations. Epilepsia. 2011;52(7):1251-7.

40. Tan Y, Hou M, Ma S, Liu P, Xia S, Wang Y, et al. Chinese cases of early infantile epileptic encephalopathy: a novel mutation in the PCDH19 gene was proved in a mosaic male- case report. BMC Medical Genetics. 2018;19(1):92.

41. Terracciano A, Specchio N, Darra F, Sferra A, Bernardina BD, Vigevano F, et al. Somatic mosaicism of PCDH19 mutation in a family with low-penetrance EFMR. Neurogenetics. 2012;13(4):341-5.

42. Terracciano A, Trivisano M, Cusmai R, De Palma L, Fusco L, Compagnucci C, et al. PCDH19-related epilepsy in two mosaic male patients. Epilepsia. 2016;57(3):e51-5.

43. Thiffault I, Farrow E, Smith L, Lowry J, Zellmer L, Black B, et al. PCDH19-related epileptic encephalopathy in a male mosaic for a truncating variant. Am J Med Genet A. 2016;170(6):1585-9.

44. Trivisano M, Lucchi C, Rustichelli C, Terracciano A, Cusmai R, Ubertini GM, et al. Reduced steroidogenesis in patients with PCDH19-female limited epilepsy. Epilepsia. 2017;58(6):e91-e5.

45. Trivisano M, Pietrafusa N, Terracciano A, Marini C, Mei D, Darra F, et al. Defining the electroclinical phenotype and outcome of PCDH19-related epilepsy: A multicenter study. Epilepsia. 2018;59(12):2260-71.

46. van Harssel JJ, Weckhuysen S, van Kempen MJ, Hardies K, Verbeek NE, de Kovel CG, et al. Clinical and genetic aspects of PCDH19-related epilepsy syndromes and the possible role of PCDH19 mutations in males with autism spectrum disorders. Neurogenetics. 2013;14(1):23-34.

47. Vincent AK, Noor A, Janson A, Minassian BA, Ayub M, Vincent JB, et al. Identification of genomic deletions spanning the PCDH19 gene in two unrelated girls with intellectual disability and seizures. Clin Genet. 2012;82(6):540-5.

48. Vlaskamp DRM, Bassett AS, Sullivan JE, Robblee J, Sadleir LG, Scheffer IE, et al. Schizophrenia is a later-onset feature of PCDH19 Girls Clustering Epilepsy. Epilepsia. 2019;60(3):429-40.

49. Yang L, Liu J, Su Q, Li Y, Yang X, Xu L, et al. Novel and de novo mutation of PCDH19 in Girls Clustering Epilepsy. Brain and Behavior. 2019;9(12):e01455.

50. Zhang X, Chen N, Ma A, Wang X, Sun W, Gao Y. Case report of a novel PCDH19 frameshift mutation in a girl with epilepsy and mental retardation limited to females. Medicine. 2018;97(51).

51. Zhao X, Wang Y, Mei S, Kong X. A novel PCDH19 missense mutation, c.812G>A (p.Gly271Asp), identified using whole-exome sequencing in a Chinese family with epilepsy female restricted mental retardation syndrome. Molecular Genetics & Genomic Medicine. 2020;8(6):e1234.
